# Risk-Appropriate Childbirth Care Among Higher-Risk Pregnant Rural Residents

**DOI:** 10.1001/jamahealthforum.2025.4241

**Published:** 2025-11-21

**Authors:** Sara C. Handley, Brielle Formanowski, Molly Passarella, Maggie L. Thorsen, Julia D. Interrante, Clara E. Busse, Scott A. Lorch, Katy B. Kozhimannil

**Affiliations:** 1 Division of Neonatology, The Children’s Hospital of Philadelphia, Philadelphia, Pennsylvania; 2 Department of Pediatrics, Perelman School of Medicine at the University of Pennsylvania, Philadelphia; 3 Leonard Davis Institute of Health Economics, University of Pennsylvania, Philadelphia; 4 Department of Sociology and Anthropology, Montana State University, Bozeman; 5 Division of Health Policy and Management, University of Minnesota School of Public Health, Minneapolis

## Abstract

**Importance:**

With hospital-based obstetric care declining in rural areas, risk-appropriate care, which aligns patient clinical conditions with hospital capabilities using level of care, may be limited for pregnant rural residents, especially those with higher-risk conditions that necessitate specialty or subspecialty obstetric care.

**Objective:**

To assess the proportion of higher-risk pregnant rural residents who receive risk-appropriate care during childbirth and identify factors associated with not receiving risk-appropriate care.

**Design, Setting, and Participants:**

This cross-sectional study used linked vital statistics and hospital discharge data for pregnant rural residents with higher-risk clinical conditions who had hospital-based births in Michigan (2010-2020), Oregon (2010-2020), Pennsylvania (2010-2018), and South Carolina (2010-2020). Data analyses were performed between December 2023 and July 2025.

**Exposure:**

Birth hospital maternal level of care (I, basic; II, specialty; III, subspeciality; IV, regional perinatal).

**Main Outcomes and Measures:**

The main outcome was birth in a hospital with risk-appropriate care, defined as having the necessary level of care for the patient’s clinical condition. Covariates included age, race and ethnicity, insurance, education, prenatal care utilization, medical and obstetric comorbidities, distance to the closest risk-appropriate hospital (quartile 1: 0.50-5.57 miles, quartile 2: 5.58-18.90 miles, quartile 3: 18.91-33.93 miles, quartile 4: 33.94-209.80 miles), year, and state.

**Results:**

A total of 199 225 higher-risk pregnant rural residents (mean [SD] maternal age, 27.9 [5.6] years) were included, of whom 11 651 (5.9%) identified as Hispanic, 3054 (1.5%) as non-Hispanic American Indian or Alaska Native, 1370 (0.7%) as non-Hispanic Asian or Pacific Islander, 18 296 (9.2%) as non-Hispanic Black, 5320 (2.7%) as non-Hispanic other race, and 159 253 (79.9%) as non-Hispanic White. Birth at a risk-appropriate hospital occurred for 38 441 of 70 647 individuals (54.4%) with conditions requiring level II care, 4611 of 9270 (49.7%) with conditions requiring level III care, and 1793 of 6527 (27.5%) with conditions requiring level IV care. Those with significantly higher rates of not receiving risk-appropriate care included American Indian or Alaska Native (adjusted incidence rate ratio [aIRR], 1.13; 95% CI, 1.10-1.17), or Hispanic (aIRR, 1.06; 95% CI, 1.03-1.08) individuals (compared with White individuals), those without private insurance (public: aIRR, 1.03; 95% CI, 1.01-1.04; uninsured: aIRR, 1.07; 95% CI, 1.01-1.14), those who were younger and had less education (age <20 years: aIRR, 1.05; 95% CI, 1.03-1.08, compared with 30-34 years; some high school: aIRR, 1.04; 95% CI, 1.03-1.06, compared with high school degree), and those who lived further from a risk-appropriate hospital (furthest quartile: aIRR, 23.86; 95% CI, 20.48-27.79, compared with closest quartile).

**Conclusions and Relevance:**

In this study, lack of risk-appropriate care was common for pregnant rural residents with clinical complexity. Associated factors, including race, ethnicity, insurance, age, education, and distance, highlight the barriers and need for increasing access to subspecialty care for pregnant rural residents.

## Key Points

**Question:**

What proportion of pregnant rural residents receive risk-appropriate childbirth care, and what factors are associated with not receiving risk-appropriate care?

**Findings:**

In this cross-sectional study of nearly 200 000 pregnant rural residents with higher-risk conditions, as clinical complexity increased, the proportion receiving risk-appropriate care decreased. Identifying as American Indian or Alaska Native or as Hispanic, being younger, having lower educational attainment, having public insurance or no insurance, and living further from a risk-appropriate hospital were factors significantly associated with not receiving risk-appropriate care.

**Meaning:**

These findings highlight the need for reducing distance to local childbirth care and increasing access to subspecialty care for pregnant rural residents.

## Teaser

This cross-sectional study assesses the proportion of higher-risk pregnant rural residents in the US who receive risk-appropriate childbirth care and identifies facrots associated with not receiving risk-appropriate care.

## Introduction

Twenty percent of people in the US live in rural areas, including millions of pregnant residents.^1^ Compared with those in urban areas, rural residents experience higher rates of death and disease, including pre-existing and pregnancy-associated conditions among pregnant residents.^2,3,4,5^ Despite this, access to hospital-based obstetric care in rural areas is limited and declining.^6,7^ Maintaining essential obstetric staff, equipment, and services at rural hospitals is challenging and crucial to high-quality obstetric care.^8,9,10^ Limited availability of hospital-based obstetric care and associated services are exacerbated for those with higher-risk conditions that necessitate specialized obstetric care.^11,12^ These challenges compound inequities in obstetric outcomes for pregnant rural residents.^13,14^

Aligning the clinical needs of pregnant patients with hospital resources and capabilities defines risk-appropriate care.^15^ In obstetrics, risk-appropriate care is operationalized through guidelines describing levels of maternal care, with level I offering basic care, level II specialty care, level III subspecialty care, and level IV high acuity regional perinatal care.^11^ Achieving risk-appropriate care is multifactorial and requires accurate prenatal diagnosis of clinical conditions, timely completion of prenatal screening, monitoring responses to clinical treatments, proper referral and access to specialists and subspecialists, and transfer to higher-level care when emergent conditions occur.^16^ Population-based data suggest that pregnant patients with complex comorbidities, who are most likely to require higher-level risk-appropriate care, are less likely to give birth in hospitals with risk-appropriate care.^12,17^ However, this question has not been examined with attention to the elevated risks and decreased access faced by pregnant rural residents. Thus, the study objectives were to (1) assess the proportion of pregnant rural residents with higher-risk conditions who received risk-appropriate care during the childbirth hospitalization and (2) identify factors associated with not receiving risk-appropriate care.

## Methods

### Study Design, Data, and Population

Using linked vital statistics (birth and death certificates) and inpatient administrative hospital discharge data, we performed a cross-sectional study examining births among pregnant rural residents across 4 states: Michigan (2010-2020), Oregon (2010-2020), Pennsylvania (2010-2018), and South Carolina (2010-2020). These 4 states were selected given the availability of linked, comprehensive data with respect to region, rurality, and sociodemographic composition. Maternal and infant data were obtained from the respective state health departments and linked by each state prior to the study team’s access. The inpatient administrative dataset contains patient sociodemographic data, hospital diagnoses, and relevant *International Classification of Diseases, Ninth Revision (ICD-9)* and *International Statistical Classification of Diseases and Related Health Problems, Tenth Revision (ICD-10)* codes.

The study population was pregnant rural residents with higher-risk conditions. We identified rural residents using 2013 Urban Influence Codes [UICs] and included those living in nonmetropolitan counties (UICs 3-12).^18^ We identified higher-risk pregnant patients if they had a medical or obstetric condition necessitating a specific level of maternal care, I through IV, as per the approach published by Easter et al^12^ (eTable 1 in Supplement 1). Study population identification is depicted in eFigure 1 in Supplement 1.

This study followed the Strengthening the Reporting of Observational Studies in Epidemiology (STROBE) reporting guideline and was reviewed and approved by the Institutional Review Board at the Children’s Hospital of Philadelphia and the Human Research Committees in the states that provided data. The need for informed consent was waived under 45 CFR 46 (the Common Rule).

### Outcome and Variables

The primary outcome was childbirth at a hospital with an appropriate level of maternal care for the patient’s clinical needs. We defined birth at a risk-appropriate hospital as having the necessary level of care or higher for the patient’s condition (eg, a patient requiring level II care receives risk-appropriate care in a hospital with level II, III, or IV maternal care). We operationalized this definition based on the single condition warranting the highest level of care (eg, a pregnant patient with 2 clinical conditions, one warranting level II care and one level III care, is risk-appropriate for level III care). Determination of the conditions and associated appropriate maternal level of care were based on published work by Easter et al^12^ and national guidelines from the American College of Obstetricians and Gynecologists/Society of Maternal-Fetal Medicine (ACOG/SMFM) for basic care (level I; eg, gestational diabetes), specialty care (level II; eg, preterm multiple gestation), subspecialty care (level III; eg, chronic kidney disease), and regional perinatal health care (level IV; eg, congestive heart failure).^12^

To determine the birth hospital level of care, we applied a published empiric approach.^19^ Based on the ACOG/SMFM level of care guidelines, this hierarchical approach uses *ICD-9* and *ICD-10* codes from the administrative data to assign a level of care annually for each hospital, independent of hospital self-report. The maternal level of care assignment was automated and output reviewed independently by 2 of us (S.C.H. and S.A.L), with discussion of final level of care assignment when reviews differed. Publicly reported and verified maternal levels of care are not available in these 4 states.

The following were included as covariates: age (increasing maternal age is associated with obstetric comorbid conditions)^20^; race and ethnicity (included as a proxy for structural racism and documented differences in obstetric outcomes and care, based on birth certificate parental report, with those selecting more than 2 races categorized as “Other”)^21^; insurance type (publicly insured pregnant patients have higher morbidity rates)^22^; educational attainment (lower educational attainment is associated with higher rates of adverse obstetric outcomes)^23^; Kotelchuck index (measures adequacy of prenatal care utilization)^24^; birth state (given differences in perinatal health systems)^6^; and year. We examined medical and obstetric comorbidities associated with adverse outcomes or a specific level of maternal care, which included chronic hypertension, hypertensive disorders of pregnancy, pregestational or gestational diabetes, obesity (>40 body mass index [calculated as weight in kilograms divided by height in meters squared] at the time of birth), bleeding disorders, asthma, severe cardiac conditions, human immune-deficiency virus, substance use disorder, chronic kidney disease, placenta previa, multiple gestation, and preterm birth.^12,25^ We examined distance using geographic location of the population-weighted centroid ZIP code tabulated area of the pregnant rural resident’s ZIP code of residence and hospital addresses to calculate the most direct distances from the pregnant rural resident to the hospital where the birth occurred, the closest birth hospital, and the closest risk-appropriate birth hospital.

### Statistical Analysis

Multivariable analyses examined whether rural pregnant residents gave birth at a hospital with the required maternal level of care. We reviewed descriptive data, examining the proportion of pregnant rural residents who gave birth in a hospital with a risk-appropriate maternal level of care. Multivariable modified Poisson models with robust standard errors were constructed to identify factors associated with not receiving risk-appropriate care.^26^ A 2-sided *P* < .05 indicated statistical significance. We examined the combined association of sociodemographic and clinical characteristics, quartiles of distance to the closest risk-appropriate hospital (quartile 1: 0.50-5.57 miles, quartile 2: 5.58-18.90 miles, quartile 3: 18.91-33.93 miles, and quartile 4: 33.94-209.80 miles), state, and year. Given that hospitals with higher level care are often located in metropolitan areas, we conducted a sensitivity analysis with models stratified by rural residence in a county adjacent (UIC 3-7) or nonadjacent (UIC 8-12) to a metropolitan county. Data were analyzed from December 2023 to July 2025 using Stata, version 18 (StataCorp LLC).

## Results

**Figure 1.**
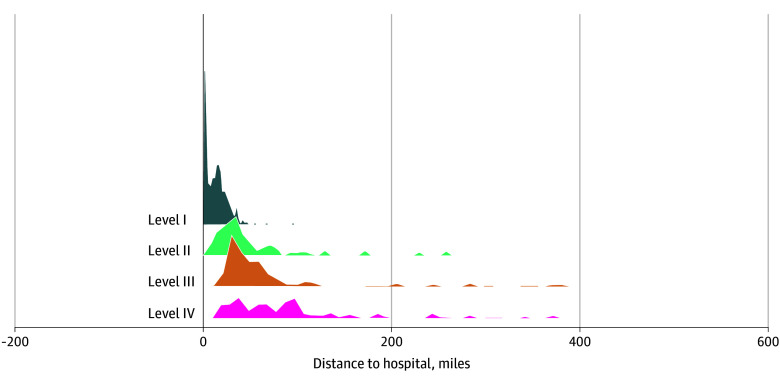
Distance to Closest Risk-Appropriate Hospital for Pregnant Rural Residents The 4 levels of maternal care include basic care (level I), specialty care (level II), subspecialty care (level III), and regional perinatal care (level IV).

The analysis included 199 225 higher-risk pregnant rural residents who gave birth at 425 hospitals, with a mean (SD) age of 27.9 (5.6) years, and of whom 11 651 (5.9%) identified as Hispanic, 3054 (1.5%) as non-Hispanic American Indian or Alaska Native, 1370 (0.7%) as non-Hispanic Asian or Pacific Islander, 18 296 (9.2%) as non-Hispanic Black, 5320 (2.7%) as non-Hispanic other race, and 159 253 (79.9%) as non-Hispanic White. In the cohort, 112 781 births (56.6%) were risk-appropriate for level I, 70 647 (35.5%) risk-appropriate for level II, 9270 (4.7%) risk-appropriate for level III, and 6527 (3.3%) risk-appropriate for level IV (Table). Distance to the closest hospital with risk-appropriate care increased substantially with each higher level of care (Figure 1), with median distances for those risk-appropriate for level I care of 10.7 miles (IQR, 2.8-19.2 miles) and those risk-appropriate for level IV of 66.1 miles (IQR, 40.7-113.4 miles) (Table).

**Table.** Pregnant Rural Resident Characteristics by Risk-Appropriate Level of Maternal Care for Childbirth

Characteristic^a^	Risk appropriate for level I (n = 112 781)	Risk appropriate for level II (n = 70 647)	Risk appropriate for level III (n = 9270)	Risk appropriate for level IV (n = 6527)
**Sociodemographic characteristics, No. (column %)**				
Maternal age, y				
<20	5181 (4.6)	5068 (7.2)	622 (6.7)	368 (5.6)
20-29	61 455 (54.5)	42 508 (60.2)	5123 (55.3)	3675 (56.3)
30-34	29 689 (26.3)	15 052 (21.3)	2178 (23.5)	1615 (24.7)
35-39	13 463 (11.9)	6546 (9.3)	1057 (11.4)	701 (10.7)
40-44	2821 (2.5)	1390 (2.0)	268 (2.9)	152 (2.3)
≥45	172 (0.15)	83 (0.12)	22 (0.24)	16 (0.25)
Maternal race and ethnicity^b^				
Hispanic	7491 (6.6)	3228 (4.6)	443 (4.8)	489 (7.5)
Non-Hispanic American Indian or Alaska Native	1379 (1.2)	1442 (2.0)	128 (1.4)	105 (1.6)
Non-Hispanic Asian or Pacific Islander	986 (0.87)	280 (0.40)	61 (0.66)	43 (0.66)
Non-Hispanic Black	10 959 (9.7)	5522 (7.8)	1419 (15.3)	396 (6.1)
Non-Hispanic, other race	2757 (2.4)	2133 (3.0)	261 (2.8)	169 (2.6)
Non-Hispanic White	89 058 (79.0)	57 935 (82.0)	6944 (74.9)	5316 (81.5)
Maternal insurance^c^				
Government	52 846 (46.9)	44 255 (62.6)	5155 (55.6)	3316 (50.8)
Private	57 754 (51.2)	25 125 (35.6)	3978 (42.9)	3101 (47.5)
Other	535 (0.47)	490 (0.69)	40 (0.43)	61 (0.93)
Self-pay	1646 (1.5)	777 (1.1)	97 (1.1)	49 (0.75)
Education attained^d^				
No high school	2757 (2.4)	1341 (1.9)	161 (1.7)	136 (2.1)
Some high school	11 518 (10.2)	12 121 (17.2)	1156 (12.5)	736 (11.3)
High school degree	33 949 (30.1)	25 814 (36.5)	3084 (33.3)	2009 (30.8)
Some college	40 444 (35.9)	22 460 (31.8)	3294 (35.5)	2273 (34.8)
4 y College	16 328 (14.5)	5972 (8.5)	1071 (11.6)	927 (14.2)
>4 y College	7509 (6.7)	2715 (3.8)	474 (5.1)	426 (6.5)
**Medical and obstetric characteristics, No. (column %)**				
Chronic hypertension	5757 (5.1)	4166 (5.9)	1850 (20.0)	366 (5.6)
Hypertensive disorder of pregnancy	15 446 (13.7)	4482 (6.3)	467 (5.0)	327 (5.0)
Pregestational or gestational diabetes	22 225 (19.7)	9441 (13.4)	1537 (16.6)	770 (11.8)
Obesity (>40 BMI at time of birth)	20 572 (18.2)	11 134 (15.8)	1870 (20.2)	921 (14.1)
Bleeding disorder	0	5905 (8.4)	411 (4.4)	201 (3.1)
Asthma	0	17 478 (24.7)	904 (9.8)	568 (8.7)
Severe cardiac condition	0	0	0	1113 (17.1)
HIV	0	0	191 (2.1)	DS
Substance use disorder	0	31 868 (45.1)	966 (10.4)	706 (10.8)
Chronic kidney disease	0	0	1099 (11.9)	82 (1.3)
Placenta previa	0	1340 (1.9)	224 (2.4)	33 (0.5)
Kotelchuck Index^e^				
Inadequate	12 405 (11.0)	10 873 (15.4)	1255 (13.5)	739 (11.3)
Intermediate	11 218 (10.0)	7658 (10.8)	654 (7.1)	599 (9.2)
Adequate	42 882 (38.0)	22 841 (32.3)	2046 (22.1)	2157 (33.1)
Adequate plus	43 077 (38.2)	26 767 (37.9)	4936 (53.3)	2795 (42.8)
Missing	3199 (2.8)	2508 (3.6)	379 (4.1)	237 (3.6)
Multiple gestation	4512 (4.0)	7431 (10.5)	980 (10.6)	412 (6.3)
Preterm birth (<37 wk)	16 379 (14.5)	15 484 (21.9)	6026 (65.0)	1419 (21.7)
Birth hospital proximity, median (IQR), mi				
Distance to the closest birth hospital, mi	10.7 (2.8-19.2)	11.3 (2.8-20.0)	10.7 (2.9-19.9)	10.2 (2.7-19.0)
Distance to closest risk-appropriate birth hospital, mi	10.7 (2.8-19.2)	31.6 (20.0-51.8)	52.9 (34.9-75.5)	66.1 (40.7-113.4)

Abbreviations: BMI, body mass index (calculated as weight in kilograms divided by height in meters squared); HIV, human immunodeficiency virus; DS, data suppressed.

^a^ All characteristics significantly differed across risk-appropriate groups (*P* values all <.001).

^b^ Race was missing for 281 births (0.14%). Individuals who select multiple races (if possible in their state) are included in the non-Hispanic Other group.

^c^ Maternal insurance categories included government (inclusive of Medicaid and Tricare), private, other, and uninsured (inclusive of self-pay).

^d^ Maternal education was missing for 550 births (0.28%).

^e^ The Kotelchuck Index classifies adequacy of prenatal care based on the expected number of visits for the period from when prenatal care began until the date of childbirth (inadequate: <50% of expected visits; intermediate: 50%-79% of expected visits; adequate: 80%-109% of expected visits; adequate plus: 110% of expected visits).

With respect to where births happened, 92 323 (45.8%) occurred at a hospital with level I care, 58 312 (29.3%) with level II, 12 431 (6.2%) with level III, and 37 159 (18.7%) with level IV (eTable 2 in Supplement 1). Rural residents who were 35 years or older, had private insurance, and had higher educational attainment more commonly gave birth in hospitals with higher-level (III or IV) care. Rural residents who were non-Hispanic American Indian or Alaska Native or Hispanic and those with public insurance were more likely to give birth at hospitals with lower levels (I or II) of care. The median distance to the hospital where birth occurred was longer among those who gave birth in hospitals with higher levels of care (9.2 miles [IQR, 2.5-19.9 miles] for those with level I maternal care compared with 39.6 miles [IQR, 26.7-60.7 miles] for those with level IV maternal care) (eTable 2 in Supplement 1).

**Figure 2.**
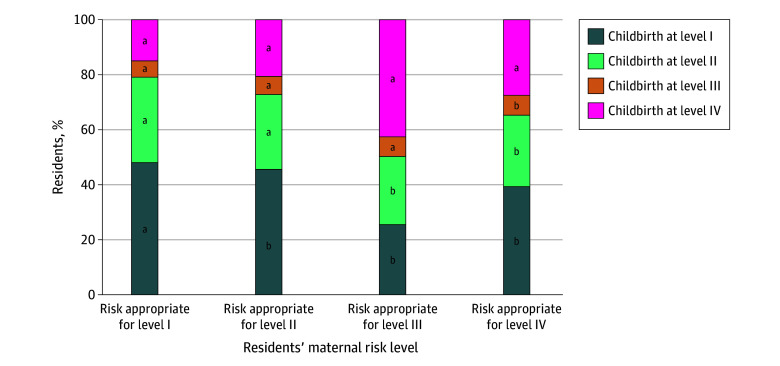
Receipt of Risk-Appropriate or Risk-Inappropriate Care Based on Maternal Risk Among Pregnant Rural Residents Maternal risk-appropriate level of care was determined using the diagnoses and associated codes described by Easter et al.^12^ ^a^Risk-appropriate care at the time of childbirth. ^b^Risk-inappropriate care at the time of childbirth.

Across the cohort, 157 626 (79.1%) of higher-risk rural residents received risk-appropriate care. As pregnant rural resident clinical complexity increased, the proportion receiving risk-appropriate care decreased; 38 441 (54.4%) of those with conditions requiring level II care, 4611 (49.7%) of those with conditions requiring level III care, and 1793 (27.5%) of those with conditions requiring level IV care gave birth at a risk-appropriate hospital (Figure 2).

**Figure 3.**
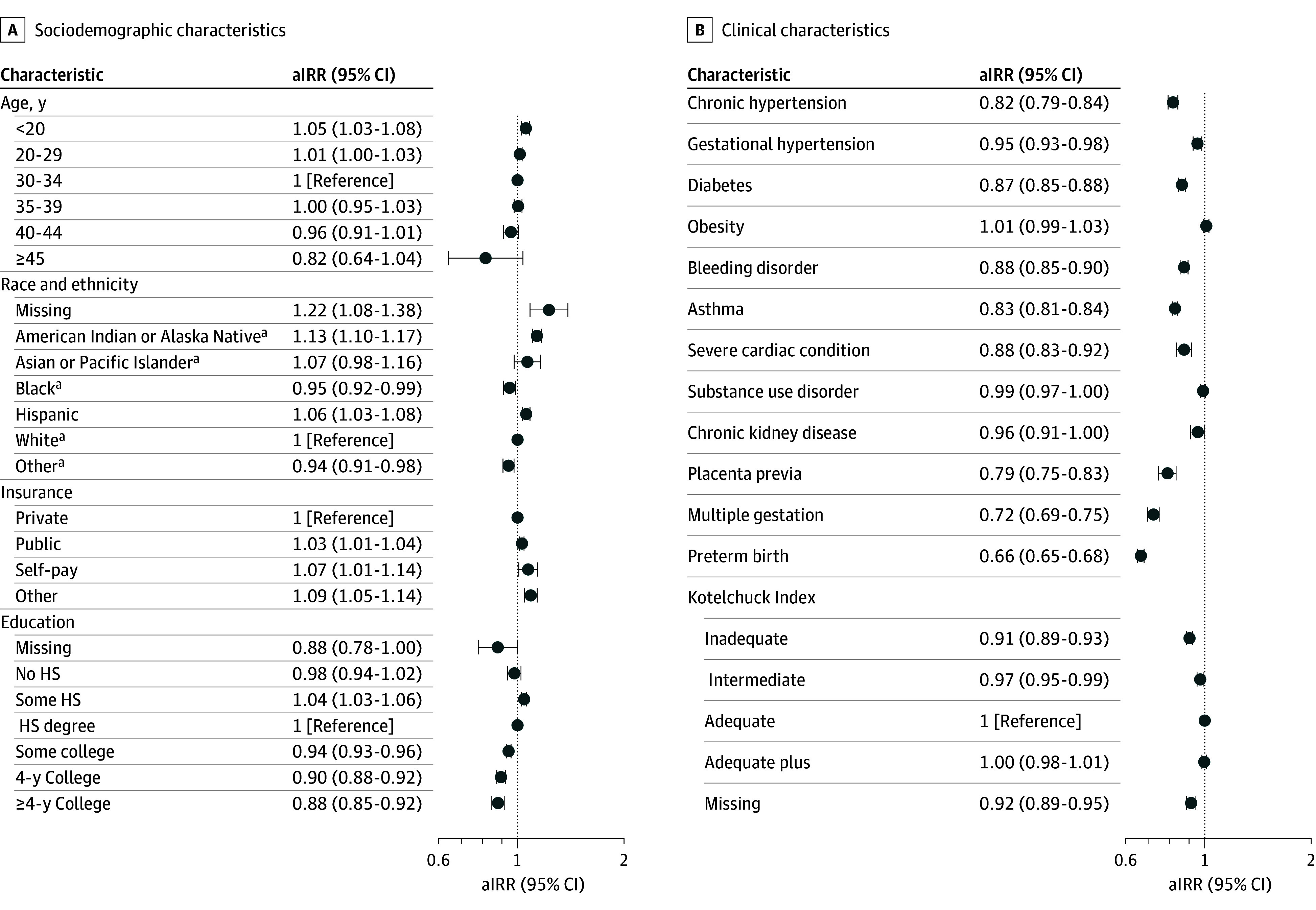
Sociodemographic and Clinical Characteristics Associated With Rural Residents Not Receiving Risk-Appropriate Care The multivariable model included all sociodemographic characteristics, clinical characteristics, and distance (closest risk-appropriate hospital quartile). aIRR indicates adjusted incidence rate ratio; HS, high school. ^a^Not Hispanic.

**Figure 4.**
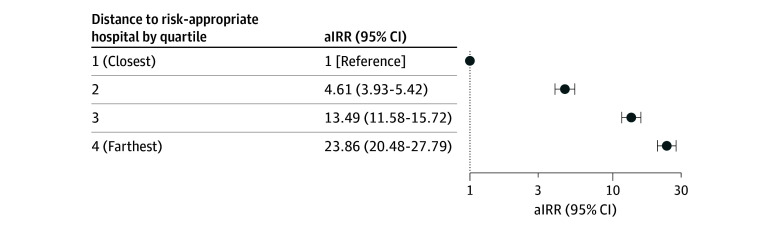
Association of Distance to Closest Risk-Appropriate Birth Hospital With Rural Residents Not Receiving Risk-Appropriate Care The distance to the closest hospitals with risk-appropriate care increases by quartile; quartile 1: 0.50-5.57 miles (reference), quartile 2: 5.58-18.90 miles, quartile 3: 18.91-33.93 miles, and quartile 4: 33.94-209.80 miles. The multivariable model included all sociodemographic characteristics, clinical characteristics, and distance (closest risk-appropriate hospital quartile). aIRR indicates adjusted incidence rate ratio.

The multivariable model revealed that patients who were younger than 20 years (adjusted incidence rate ratio [aIRR], 1.05; 95% CI, 1.03-1.08; reference, 30-34 years), who identified as non-Hispanic American Indian or Alaska Native (aIRR, 1.13; 95% CI, 1.10-1.17) or Hispanic (aIRR, 1.06; 95% CI, 1.03-1.08; reference, non-Hispanic White), had nonprivate insurance (public: aIRR, 1.03, 95% CI 1.01-1.04; uninsured: aIRR, 1.07; 95% CI, 1.01-1.14; reference, private), or some high school education (aIRR, 1.04; 95% CI, 1.03-1.06; reference, high school degree) all had higher rates of birth in a hospital without risk-appropriate care (Figure 3A). Except for the presence of obesity, substance use disorder, and chronic kidney disease diagnoses, which were nonsignificant, the other clinical diagnoses studied were significantly associated with receiving risk-appropriate care (Figure 3B). Figure 4 depicts the aIRR for distance to the closest risk-appropriate hospital by quartile, with the rates of not receiving risk-appropriate care increasing significantly with longer distances (furthest quartile: aIRR, 23.86; 95% CI, 20.48-27.79; reference, closest quartile). Results of the sensitivity analysis stratified by adjacency to a metropolitan county were similar to the primary analysis results (eTable 3 in Supplement 1).

## Discussion

This cross-sectional study suggests that for pregnant rural residents, especially those with the highest-risk clinical conditions who live long distances from hospitals with subspecialty services, accessing risk-appropriate hospital-based care for childbirth is a challenge. The mismatch between clinical needs and hospital capabilities is amplified for pregnant rural residents with the most complex clinical conditions, with only one-quarter of those who warrant level IV care ultimately giving birth in a risk-appropriate hospital. Even for those rural residents who needed level II maternal care, only half had risk-appropriate services available where they gave birth. One of the most notable barriers was longer distance to risk-appropriate care, a particular challenge faced by pregnant rural residents.

After controlling for sociodemographic and clinical characteristics as well as distance to care, this analysis revealed some of the disparities associated with lack of risk-appropriate care. While the presence of most clinical conditions were associated with a higher rate of risk-appropriate care, factors such as younger age, less educational attainment, being non-Hispanic American Indian or Alaska Native or Hispanic, having public insurance, or being uninsured were associated with significantly lower rates of risk-appropriate childbirth care. Distance was the strongest factor associated with not receiving risk-appropriate care, highlighting an access challenge that is heightened as rural hospitals continue to close obstetric units. To our knowledge, this is the first study to examine receipt of risk-appropriate care in a rural population. These data demonstrate systematic challenges facing rural residents in accessing risk-appropriate obstetric care, including distance to care, which is a distinct issue in remote and rural areas. These findings highlight potential opportunities for organizational and policy-based approaches to optimize availability and access to risk-appropriate care for rural residents.

Risk-appropriate obstetric care has been an explicit national goal since 1975.^27^ However, prior research shows that many births in the US happen in hospitals that are insufficiently equipped to meet the needs of high-risk obstetric patients.^12^ Specifically, 2014 data from 7 states found that among higher-risk patients warranting level III or IV care, 43.4% gave birth in a hospital with inappropriately lower-level care.^12^ In the current study, which used the same higher-risk patient definitions, we found 59.5% of rural residents gave birth in a hospital with inappropriately low-level care. Risk-appropriate care is relevant for both urban and rural communities, yet rural residents are more likely to give birth in hospitals without subspeciality perinatal care.^28^ Building on prior literature, these results illustrate the magnitude of this public health problem. Additionally, systematic inequities reflected in age, education, race, ethnicity, insurance, and distance to care as significant barriers to risk-appropriate care highlight the structural urbanism experienced by rural residents, as many policies are designed to address the needs of a majority urban population without considering the distinct needs of rural communities.^29^ Further, these inequities intersect; for example, pregnant Indigenous rural residents experience higher risks of severe maternal morbidity and mortality compared with their rural non-Hispanic White and urban Indigenous counterparts.^30^ Our finding that non-Hispanic American Indian or Alaska Native pregnant rural residents are less likely to receive risk-appropriate childbirth care reflect the multifaceted systems of structural racism that have disenfranchised such rural communities and undermine essential health care access.^31,32^

Given that longer distances to obstetric care are associated with adverse outcomes, strategies to improve patient triage, subspeciality consultation, regional perinatal referral and transfer systems, and patient transportation could be developed to support risk-appropriate childbirth care.^33,34^ Researchers in rural Queensland, Australia, have studied risk scores to identify and transfer patients with an increased likelihood of needing intervention during birth to higher-level care.^35,36^ Testing and standardizing similar approaches in the US may support timely identification of higher-risk patients. While the COVID-19 pandemic drove rapid adoption of telehealth services in obstetric care, an emphasis on rural-specific telehealth services predates the pandemic.^37,38^ Obstetric telehealth services have largely focused on prenatal and postpartum care, which may facilitate diagnosis of pregnancy-associated comorbidities, aid detection of postpartum complications, and address systematic disparities in access.^39,40^ Risk-appropriate obstetric care during childbirth may be improved via telehealth programs to address the geographic maldistribution of subspecialists (eg, through virtual appointments), support clinical decision-making in acute scenarios (eg, clinician-to-clinician consultation, as has been done in neonatal resuscitation), and provide simulation (eg, telesimulation).^38,41,42^ The use of telehealth services relies on broadband access, and the rural communities furthest from obstetric care often have less connectivity.^43^ Arkansas is an example of a successful telehealth program, where statewide partnerships drove broadband expansion that supported a telehealth program to provide high-risk pregnant residents with access to subspecialists, disseminate care guidelines, and facilitate referrals to higher-level care.^44^ While the number of states with maternal transport policies has increased over time, the presence of both a maternal transport policy and financial reimbursement policy (the latter being less common) remains varied across states.^45,46^ Updating policies and payment structures to incentivize hospitals to pursue risk-appropriate transfer prior to birth may optimize maternal transfers. Rural-specific triage, broadband access, telehealth services, and maternal transport policies may help address distance challenges for higher-risk pregnant rural residents.

The majority (56.6%) of higher-risk pregnant rural residents in this study had conditions risk-appropriate for level I care. This illustrates the importance of preserving local access to high-quality level I maternal care. Such preservation relies on thorough assessment of obstetric care regionalization, specifically maintaining birth hospitals in remote regions as has been done in the Portuguese Azores and studied in Finland.^47,48^ Rural hospitals in the US, including those offering obstetric services, face financial and logistical threats to maintaining services, including lower birth volumes; a high proportion of Medicaid-paid births, which have lower reimbursement rates than privately-reimbursed births; and costly malpractice insurance, all of which result in high fixed costs that volume-based payments may not adequately cover.^49,50,51^ These threats could be mitigated through policies that support standby capacity, low-volume payment adjustments, and adequate reimbursement to cover the costs of obstetric services.^51^ Advocating for the maintenance and expansion of rural-specific and Medicaid-serving programs, such as the Critical Access Hospital program and Medicaid Disproportionate Share Hospital Payments, may help financially support rural obstetric care.^52,53^ Efforts to preserve hospital-based obstetric care through thoughtful geographic distribution and policies that support rural hospital financial viability and health benefit higher-risk and low-risk pregnant rural residents alike.

### Limitations

This study has limitations. First, there are varied definitions to identify higher-risk pregnant patients, and some higher-risk patients may have been excluded based on the definition applied. While such definitions are evolving, using a previously published approach builds consistency in the literature.^12^ Second, the maternal levels of care used in this study, which were developed in the past decade and based on clinical opinion, cannot easily be validated because publicly available, national data do not exist. However, the applied approach was empiric and dynamic, reflecting the actual care hospitals provided and accounting for changes, specifically closures, which are prominent in rural areas. Third, while we included race and ethnicity, which were obtained from birth certificate data, as proxy variables for structural racism, examination of other structural factors is limited by available variables. Fourth, because this is a cross-sectional study of existing data, there is a risk of unmeasured confounding in analyses. Similarly, our analyses report associations and cannot elucidate specific mechanisms that drive differences in receiving risk-appropriate care, which are hypothesis generating. Fifth, patients in this study do not represent a national sample, and hospital discharge data for births at Indian Health Service facilities and military hospitals are not included. However, the 4 states included provide a diverse range of geography, health care systems, patient demographics, and all payor types.

## Conclusions

This cross-sectional study found that, on average, 1 in 5 higher-risk pregnant rural residents do not receive risk-appropriate childbirth care, and those who have the highest clinical risk are the least likely to receive risk-appropriate care. Factors including age, education, insurance status, race, ethnicity, and distance to care are associated with whether pregnant rural residents get the clinical care they need at the time of childbirth. These findings highlight the need for resources, policies, and systems that support both access to subspecialty care for higher-risk patients and preservation of local obstetric care for rural communities.

## Notes

Supplement 1. **eTable.** Recommended minimum level of maternal care and higher-risk medical conditions **eFigure 1.** Study cohort identification **eTable 2.** Pregnant rural resident characteristics by level of maternal care where birth occurred **eTable 3.** Sociodemographic characteristics, clinical characteristics, and distance to closest risk-appropriate birth hospital associated with rural residents not receiving risk-inappropriate care, stratified by residence in a county adjacent or non-adjacent to a metropolitan county

Supplement 2.
Data Sharing Statement
