# Supplementary material for: Risk-Appropriate Childbirth Care Among Higher-Risk Pregnant Rural Residents
Source: JAMA Health Forum. 2025 Nov 21;6(11):e254241. doi: 10.1001/jamahealthforum.2025.4241 (PMC12639487; doi:10.1001/jamahealthforum.2025.4241)
Supplement: Supplement 1. — eTable. Recommended minimum level of maternal care and higher-risk medical conditions eFigure 1. Study cohort identification eTable 2. Pregnant rural resident characteristics by level of maternal care where birth occurred eTable 3. Sociodemographic characteristics, clinical characteristics, and distance to closest risk-appropriate birth hospital associated with rural residents not receiving risk-inappropriate care, stratified by residence in a county adjacent or non-adjacent to a metropolitan county [file jamahealthforum-e254241-s001.pdf]

Supplemental Online Content

Handley SC, Formanowski B, Passarella M, et al. Risk-appropriate childbirth care among higher-risk pregnant rural residents. *JAMA Health Forum*. 2025;6(11):e254241. doi:10.1001/jamahealthforum.2025.4241

eTable. Recommended minimum level of maternal care and higher-risk medical conditions

eFigure 1. Study cohort identification

eTable 2. Pregnant rural resident characteristics by level of maternal care where birth occurred

eTable 3. Sociodemographic characteristics, clinical characteristics, and distance to closest risk-appropriate birth hospital associated with rural residents not receiving risk-inappropriate care, stratified by residence in a county adjacent or non-adjacent to a metropolitan county

This supplemental material has been provided by the authors to give readers additional information about their work.

© 2025 Handley SC et al. JAMA Health Forum.

eTable. Recommended minimum level of maternal care and higher-risk medical conditions

Recommended minimum maternal level of care	Associated diagnoses
I	Preterm, Multiple gestation, Previous cesarean delivery, prior uterine surgery, gestational hypertension, preeclampsia without severe features, chronic hypertension, gestational diabetes
II	Preterm multiple gestation, Pre-existing diabetes mellitus, Alcohol abuse, Drug abuse, Asthma, History of DVT or PE, Hypercoagulable state, Coagulation disorder, Abruption, Preterm chronic hypertension, Preterm gestational hypertension, Preterm preeclampsia without severe features, Placenta previa
III	Previa with prior uterine surgery, Preterm preeclampsia with severe features, Chronic renal disease, Cystic fibrosis, Epilepsy, HIV, Systemic lupus erythematosus, Sickle cell disease, Hematologic cancer, Breast cancer, Maternal cancer, Gynecologic cancer
IV	Congenital heart disease, Chronic ischemic heart disease, Cardiac valvular disease, Endocarditis, Aortic Stenosis, Mitral stenosis, Aortic and mitral stenosis, Mechanical heart valve, Hypertrophic cardiomyopathy, Congestive heart failure, Marfan syndrome, Aortic aneurysm, Pulmonary hypertension, Hypoplastic left heart syndrome.

List of conditions as published by Easter SR et al. *Obstet Gynecol* 2019; 134.

© 2025 Handley SC et al. JAMA Health Forum.

eFigure 1. Study Cohort Identification

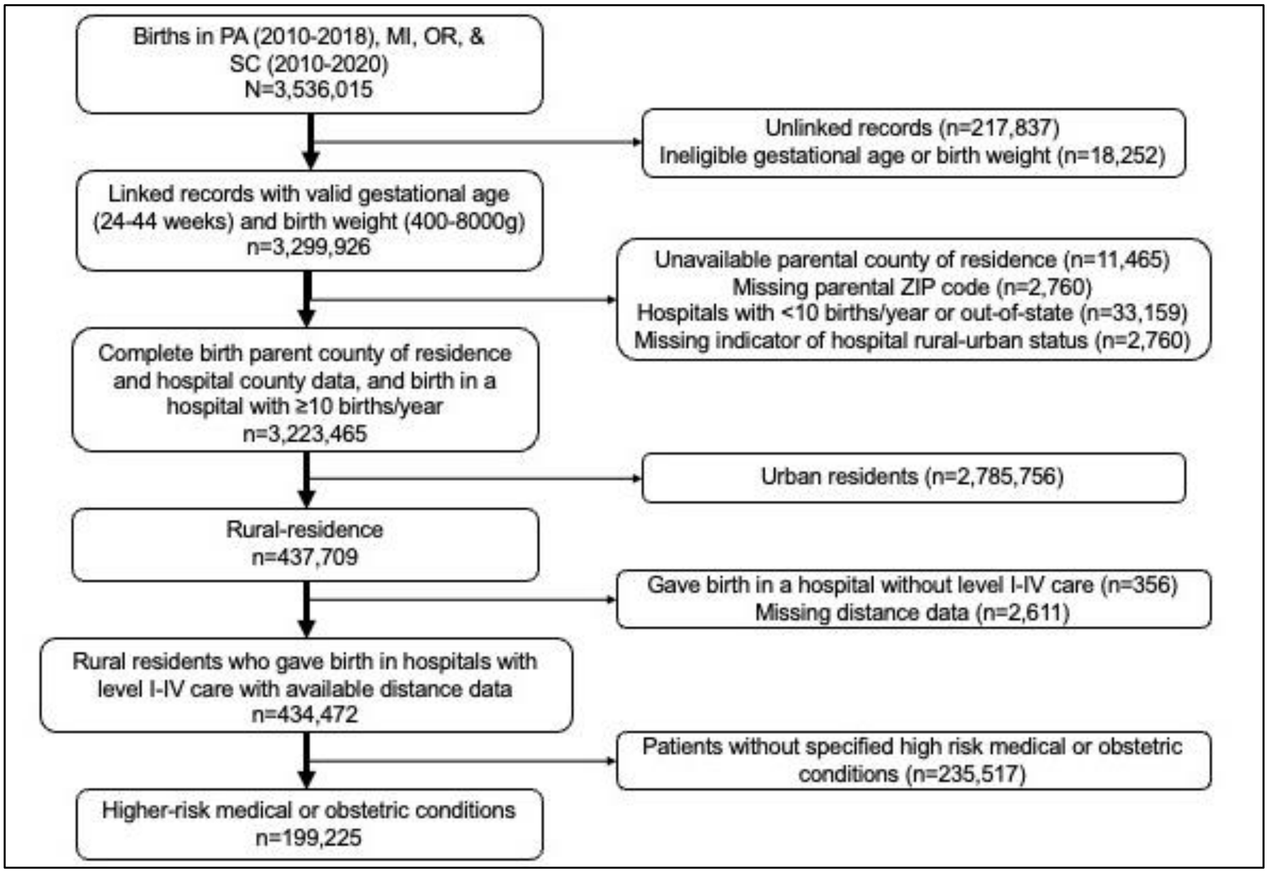

© 2025 Handley SC et al. JAMA Health Forum.

eTable 2. Pregnant rural resident characteristics by level of maternal care where birth occurred

**Sociodemographic, medical, and obstetric characteristics[a]**	**I n=91,323**	**II n=58,312**	**III n=12,431**	**IV n=37,159**
Sociodemographic characteristics, n (column %)				
Age, years				
<20	5,712 (6.3)	3,219 (5.5)	479 (3.9)	1,829 (4.9)
20-29	53,474 (58.6)	33,219 (57.0)	6,210 (50.0)	19,858 (53.4)
30-34	20,935 (22.9)	14,235 (24.4)	3,582 (28.8)	9,782 (26.3)
35-39	9,179 (10.1)	6,312 (10.8)	1,762 (14.2)	4,514 (12.2)
40-44	1,928 (2.1)	1,240 (2.1)	377 (3.0)	1,086 (2.9)
45+	95 (0.1)	87 (0.2)	21 (0.2)	90 (0.2)
Race and ethnicity[b]				
Hispanic	6,800 (7.5)	2,721 (4.7)	467 (3.8)	1,663 (4.5)
Non-Hispanic American Indian/Alaska Native	2,059 (2.3)	521 (0.9)	195 (1.6)	279 (0.8)
Non-Hispanic Asian/Pacific Islander	748 (0.8)	316 (0.5)	70 (0.6)	236 (0.6)
Non-Hispanic Black	4,986 (5.5)	8,670 (14.9)	189 (1.5)	4,451 (12.0)
Non-Hispanic Other	2,445 (2.7)	1,510 (2.6)	237 (1.9)	1,128 (3.0)
Non-Hispanic White	74,123 (81.2)	44,510 (76.3)	11,259 (90.6)	29,361 (79.0)
Insurance[c]				
Private	37,237 (40.8)	27,069 (46.4)	6,880 (55.4)	18,772 (50.5)
Public	52,162 (57.1)	30,165 (51.7)	5,435 (43.7)	17,810 (47.9)
Other	674 (0.7)	269 (0.5)	17 (0.1)	166 (0.5)
Uninsured	1,250 (1.4)	809 (1.4)	99 (0.8)	411 (1.1)
Education attained[d]				
No high school	2,259 (2.5)	1,342 (2.3)	139 (1.1)	655 (1.8)
Some high school	13,470 (14.8)	7,057 (12.1)	1,091 (8.8)	3,913 (10.5)
High school degree	32,193 (35.3)	18,125 (31.1)	3,407 (27.4)	11,131 (30.0)
Some college	30,002 (32.9)	20,881 (35.8)	4,451 (35.8)	13,137 (35.4)
4 year college	9,197 (10.1)	7,318 (12.6)	2,177 (17.5)	5,606 (15.1)
>4 years college	3,953 (4.3)	3,454 (5.9)	1,146 (9.2)	2,571 (6.9)
Medical & Obstetric Characteristics, n (column %)				
Chronic hypertension	4,116 (4.5)	3,651 (6.3)	616 (5.0)	3,756 (10.1)
Hypertensive disorder of pregnancy	9,709 (10.6)	5,918 (10.2)	950 (7.6)	4,145 (11.2)
Pregestational or gestational diabetes	14,854 (16.3)	10,147 (17.4)	2,028 (16.3)	6,944 (18.7)
Obesity (>40 BMI at time of birth)	16,745 (18.3)	8,817 (15.1)	2,086 (16.8)	6,849 (18.4)
Bleeding disorder	2,714 (3.0)	1,828 (3.1)	492 (4.0)	1,483 (4.0)
Asthma	7,717 (8.5)	5,094 (8.7)	1,393 (11.2)	4,746 (12.8)
Severe cardiac condition	311 (0.3)	235 (0.4)	60 (0.5)	507 (1.4)
HIV	41 (0.04)	56 (0.1)	DS	87 (0.2)
Substance use disorder	17,766 (19.5)	8,236 (14.1)	2,000 (16.1)	5,538 (14.9)
Chronic kidney disease	413 (0.5)	239 (0.4)	99 (0.8)	430 (1.2)
Placenta previa	545 (0.6)	460 (0.8)	119 (1.0)	473 (1.3)
Kotelchuck Index, Adequacy of Prenatal Care[e]				

© 2025 Handley SC et al. JAMA Health Forum.

Sociodemographic, medical, and obstetric characteristics[a]	I n=91,323	II n=58,312	III n=12,431	IV n=37,159
Inadequate	10,614 (11.6)	7,768 (13.3)	1,133 (9.1)	5,757 (15.5)
Intermediate	9,958 (10.9)	6,140 (10.5)	1,540 (12.4)	2,491 (6.7)
Adequate	35,289 (38.6)	19,942 (34.2)	5,842 (47.0)	8,853 (23.8)
Adequate Plus	32,146 (35.2)	23,163 (39.7)	3,545 (28.5)	18,721 (50.4)
Missing	3,316 (3.6)	1,299 (2.2)	371 (3.0)	1,337 (3.6)
Multiple gestation	3,897 (4.3)	3,764 (6.5)	1,202 (9.7)	4,472 (12.0)
Preterm birth (<37 weeks)	11,969 (13.1)	10,734 (18.4)	3,091 (24.9)	13,514 (36.4)
Birth hospital proximity in miles, median (IQR)				
Distance to where birth occurred delivered	9.2 (2.5-19.9)	21.7 (9.7-34.7)	29.4 (13.4-42.4)	39.6 (26.7-60.7)
Distance to the closest birth hospital	6.7 (2.2-16.5)	13.2 (4.5-22.2)	13.4 (4.2-21.6)	14.1 (4.6-21.1)
Distance to closest risk appropriate birth hospital	19.5 (3.6-42.4)	16.7 (5.0-27.8)	18.1 (4.2-30.0)	21.6 (13.0-32.6)

Abbreviations: BMI – Body Mass Index, HIV - Human Immune-deficiency Virus, DS – Data Suppressed, IQR – Interquartile Range
[a]All characteristics significantly differed risk-appropriate groups (p-values all <0.001).
[b]Race was missing for 241 births (0.14%). Individuals who select multiple races (if possible, in their state) are included in the "Non-Hispanic Other" group.
[c]Maternal insurance categories included government (inclusive of Medicaid and Tricare), private, other, and uninsured (inclusive of self-pay).
[d]Education attained was missing for 550 births (0.28%)
[e]The Kotelchuck Index classifying adequacy of prenatal care based on the expected number of visits for the period when care began date of childbirth; Inadequate: <50% of expected visits, Intermediate: 50-79% of expected visits, Adequate: 80-109% of expected visits, Adequate Plus: 110% of expected visits.

© 2025 Handley SC et al. JAMA Health Forum.

eTable 3. Sociodemographic characteristics, clinical characteristics, and distance to closest risk-appropriate birth hospital associated with rural residents not receiving risk-inappropriate care, stratified by residence in a county adjacent or non-adjacent to a metropolitan county.

Characteristics	Adjacent to a Metropolitan County (aIRR, 95% CI)	Non-Adjacent to a Metropolitan County (aIRR, 95% CI)
Age, years		
<20	1.08 (1.04, 1.11)	1.02 (0.98, 1.05)
20-29	1.03 (1.01, 1.05)	1.00 (0.98, 1.02)
30-34	Reference	Reference
35-39	0.98 (0.95, 1.02)	1.03 (0.99, 1.06)
40-44	0.96 (0.90, 1.03)	0.96 (0.90, 1.03)
45+	0.70 (0.48, 1.02)	0.89 (0.68, 1.18)
Race and ethnicity		
Hispanic	1.13 (1.10, 1.17)	0.97 (0.93, 1.01)
Non-Hispanic American Indian/Alaska Native	1.06 (0.93, 1.20)	1.04 (0.94, 1.14)
Non-Hispanic Asian/Pacific Islander	1.18 (1.11, 1.24)	1.07 (1.04, 1.11)
Non-Hispanic Black	0.96 (0.92, 1.00)	1.04 (0.93, 1.16)
Non-Hispanic Other	0.95 (0.90, 0.99)	0.92 (0.88, 0.96)
Non-Hispanic White	Reference	Reference
Insurance		
Private	1.04 (1.02, 1.06)	0.99 (0.98, 1.02)
Public	Reference	Reference
Other	1.14 (1.09, 1.20)	1.07 (0.97, 1.18)
Uninsured	1.12 (1.04, 1.21)	1.02 (0.93, 1.13)
Education attained		
No high school	1.00 (0.95, 1.06)	0.97 (0.91, 1.04)
Some high school	1.07 (1.04, 1.09)	1.01 (0.98, 1.03)
High school degree	Reference	Reference
Some college	0.93 (0.91, 0.95)	0.97 (0.95, 0.99)
4 year college	0.85 (0.82, 0.88)	0.96 (0.93, 0.99)
>4 year college	0.85 (0.80, 0.89)	0.94 (0.89, 0.99)
Chronic hypertension	0.80 (0.77, 0.83)	0.87 (0.83, 0.91)
Hypertensive disorder of pregnancy	0.92 (0.89, 0.96)	1.02 (0.98, 1.06)
Pregestational or gestational diabetes	0.85 (0.82, 0.87)	0.90 (0.87, 0.92)
Obesity (>40 BMI at time of birth)	1.01 (0.99, 1.03)	1.01 (0.99, 1.03)
Bleeding disorder	0.82 (0.80, 0.86)	0.93 (0.90, 0.96)
Asthma	0.81 (0.80, 0.83)	0.85 (0.83, 0.87)
Severe cardiac condition	0.88 (0.82, 0.94)	0.90 (0.84, 0.97)
Substance use disorder	0.98 (0.96, 0.99)	0.98 (0.96, 0.99)
Chronic kidney disease	0.98 (0.93, 1.05)	0.94 (0.88, 0.99)
Placenta previa	0.75 (0.70, 0.81)	0.83 (0.77, 0.90)
Multiple gestation	0.70 (0.67, 0.74)	0.74 (0.67, 0.71)
Preterm birth	0.66 (0.64, 0.67)	0.69 (0.67, 0.71)
Kotelchuck Index, Adequacy of Prenatal Care[a]		
Inadequate	0.87 (0.85, 0.89)	0.97 (0.94, 0.99)
Intermediate	0.98 (0.95, 0.99)	0.95 (0.93, 0.98)
Adequate	Reference	Reference
Adequate Plus	0.95 (0.93, 0.97)	1.07 (1.05, 1.09)
Missing	0.85 (0.82, 0.89)	1.01 (0.97, 1.05)
Closest risk-appropriate hospital		

© 2025 Handley SC et al. JAMA Health Forum.

1 (Closest)	Reference	Reference
2	5.38 (4.31, 6.72)	4.20 (3.31, 5.33)
3	17.664 (14.25, 21.90)	6.29 (5.02, 7.88)
4 (Farthest)	27.04 (21.82, 33.50)	20.31 (16.36, 25.22)

Abbreviations: aIRR – Adjusted Incidence Rate Ratio, CI – Confidence Interval, BMI – Body Mass Index, HIV - Human Immune-deficiency Virus, DS – Data Suppressed, IQR – Interquartile Range

[a]The Kotelchuck Index classifying adequacy of prenatal care based on the expected number of visits for the period when care began date of childbirth; Inadequate: <50% of expected visits, Intermediate: 50-79% of expected visits, Adequate: 80-109% of expected visits, Adequate Plus: 110% of expected visits.

© 2025 Handley SC et al. JAMA Health Forum.
