# Supplementary material for: Risk-Appropriate Childbirth Care Among Higher-Risk Pregnant Rural Residents
Source: JAMA Health Forum. 2025 Nov 21;6(11):e254241. doi: 10.1001/jamahealthforum.2025.4241 (PMC12639487; doi:10.1001/jamahealthforum.2025.4241)
Supplement: Supplement 2. — Data Sharing Statement [file jamahealthforum-e254241-s002.pdf]

Data Sharing Statement

Handley. Risk-Appropriate Childbirth Care Among Higher-Risk Pregnant Rural Residents. *JAMA Health Forum*. Published November 21, 2025. doi:10.1001/jamahealthforum.2025.4241

Data
Data available: No

Additional Information
Explanation for why data not available: Data used in this analysis contain identifiable personal health information and were obtained through data use agreements with each state that prohibit any sharing of these data. Others wanting access to these data would need to obtain their own data use agreements with the individual state departments of health.
